# Biologically targeted probes for Zn^2+^: a diversity oriented modular “click-S_N_Ar-click” approach†
†Electronic supplementary information (ESI) available: Full experimental details including characterisation of all novel compounds can be found in the ESI. See DOI: 10.1039/c4sc01249f



**DOI:** 10.1039/c4sc01249f

**Published:** 2014-06-27

**Authors:** J. Pancholi, D. J. Hodson, K. Jobe, G. A. Rutter, S. M. Goldup, M. Watkinson

**Affiliations:** a School of Biological and Chemical Science , Queen Mary University of London , Mile End Road , London , E1 4NS , UK . Email: s.m.goldup@qmul.ac.uk ; Email: m.watkinson@qmul.ac.uk; b Section of Cell Biology , Division of Diabetes , Endocrinology and Metabolism , Department of Medicine , Imperial College London , London , W12 0NN , UK . Email: g.rutter@imperial.ac.uk

## Abstract

We describe a one-pot strategy for the high yielding, operationally simple synthesis of fluorescent probes for Zn^2+^ that bear biological targeting groups and exemplify the utility of our method through the preparation of a small library of sensors. Investigation of the fluorescence behaviour of our library revealed that although all behaved as expected in MeCN, under biologically relevant conditions in HEPES buffer, a plasma membrane targeting sensor displayed a dramatic switch on response to excess Zn^2+^ as a result of aggregation phenomena. Excitingly, *in cellulo* studies in mouse pancreatic islets demonstrated that this readily available sensor was indeed localised to the exterior of the plasma membrane and clearly responded to the Zn^2+^ co-released when the pancreatic beta cells were stimulated to release insulin. Conversely, sensors that target intracellular compartments were unaffected. These results demonstrate that this sensor has the potential to allow the real time study of insulin release from living cells and exemplifies the utility of our simple synthetic approach.

## Abstract 2

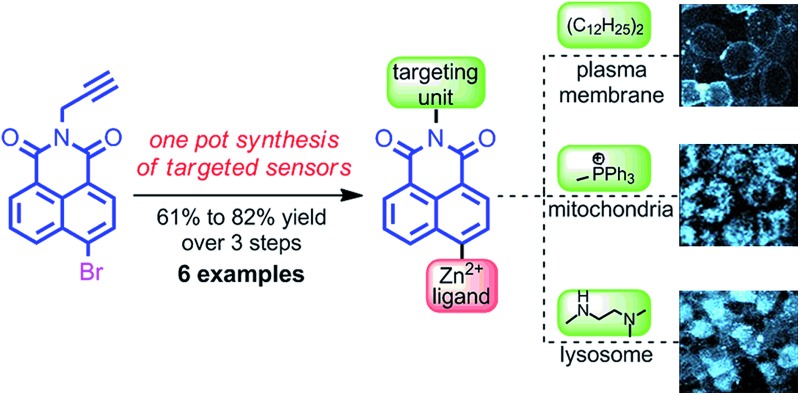
We report a high yielding, one-pot method for the synthesis of zinc responsive probes targeted to specific organelles and examine selected sensors in pancreatic islet cells.

## Introduction

Zinc is now firmly established as an essential trace element, widely found in proteins where it undertakes both structural and catalytic roles.^1^ In addition to these fixed forms of biological zinc, it is known that pools of loosely bound zinc, often referred to as “free” or “mobile”,^2^ are found in the pancreas,^3^ central nervous system,^4^ prostate,^5^ intestine^6^ and retina.^7^ The biological trafficking of zinc has been linked to many disease states in these vital organs, including Alzheimer's disease,^8^ epilepsy,^9^ ischemic stroke,^10^ infantile diarrhoea,^6^ age related macular degeneration,^7^ prostate cancer^11^ and type 2 diabetes.^3^ For example, polymorphisms in the *SLC30A8* gene, encoding the secretory granule zinc transporter ZnT8 in pancreatic islet β-cells, are directly associated with type 2 diabetes.^3^

However, although the importance of mobile zinc in human health is now well known,^12^ its role in the physiological changes leading to the onset and progress of these disease states is not yet fully understood, to a large extent due to our current inability to monitor changes in mobile zinc at high resolution in specific biological space.^13^ There is therefore a major drive to develop molecular probes which are able to map Zn^2+^ levels in cells and organs *via* the chelatable mobile pools of zinc present.

**Fig. 1:**
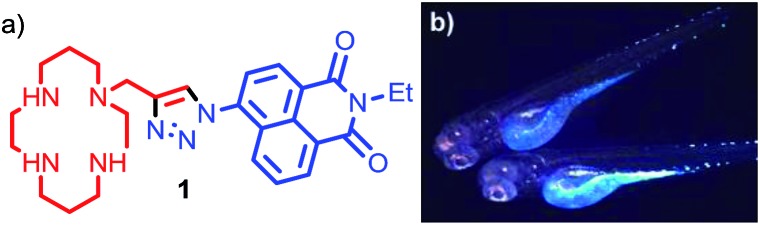
(a) Our previously reported first generation Zn^2+^ sensor **1**; (b) fluorescence microscopy images of *Danio rerio* (zebrafish) embryo *incubated* with sensor **1**.

Small molecule fluorescent probes for Zn^2+^ consisting of a chelating unit, attached (directly or indirectly) to a fluorescent moiety can deliver a rapid, strong output signal in response to binding of Zn^2+^ at the chelation site, even at very low concentrations of the metal ion.^14^ A wide range of these probes have now been reported that display an array of metal-binding and fluorescent properties and which, in principle, can be further tuned through rational synthetic modification.^15^ We previously reported probe **1**, consisting of a metal chelating tetra-azamacrocycle, linked to a fluorophore by a triazole moiety (Fig. 1a),^16^ which shows a high selectivity for Zn^2+^ over other biologically relevant metals in a suitable pH range. We demonstrated the utility of probe **1** *in vivo* in zebrafish (Fig. 1b).^17^ However, although **1** and other notable first generation small molecule Zn^2+^ probes display high selectivity for zinc,^15^ they are limited when judged against the requirements of biological imaging, in particular the need to target specific cellular and sub-cellular domains for optimal *in vivo* imaging, as well as the need to tailor the metal binding unit in order to detect mobile zinc at a wide range of biologically relevant concentrations.^14*d*^

**Fig. 2:**
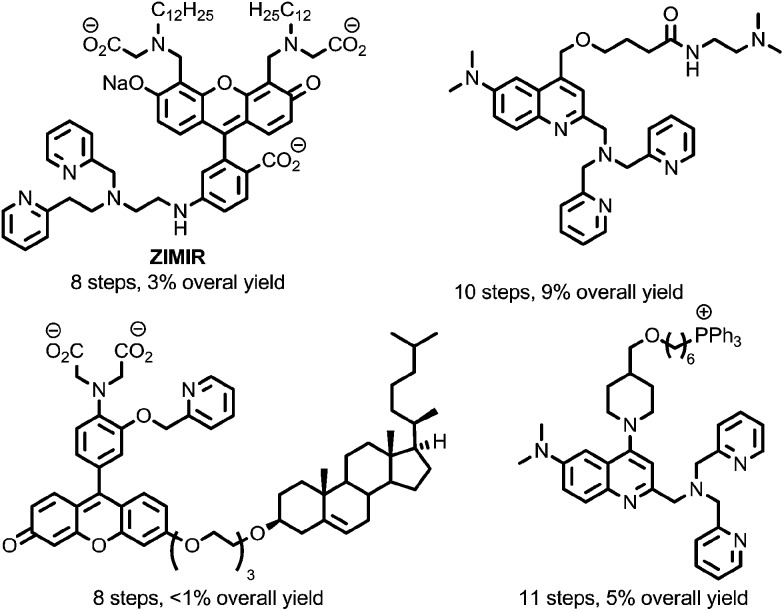
Representative examples of previously reported sensors for Zn^2+^ incorporating biological targeting units.^19^

To overcome this, a limited number of biologically targeted small molecule fluorescent probes for Zn^2+^ have been developed.^18,19^ However, these probes are typically time-consuming and challenging to prepare (Fig. 2), and this, combined with the unpredictable nature of biological systems that necessitates a heuristic approach to probe optimisation, presents a significant barrier to their application. Alternatively, targeted recombinant probes have been shown to permit measurement of Zn^2+^ at the subcellular level.^20^ However, the deployment of these probes in living cells is often limited by the need for virus-mediated or transgenic approaches to allow gene delivery.^19*c*^

To facilitate the rapid development of targeted small molecule probes for Zn^2+^ they should ideally be accessible in a synthetically flexible and succinct manner such that their properties can be optimised quickly and easily. Herein we describe such an approach; a diversity-oriented modular, one-pot synthetic method for the realisation of biologically targeted Zn^2+^ sensors and demonstrate its utility through the synthesis of a small library of sensors with a selection of metal binding and biological targeting units. The selectivity and sensitivity of the sensors were studied to confirm that the addition of the targeting moiety did not significantly alter zinc binding and fluorescent behaviour. We showcase the potential of our approach for the development of new sensors for biological applications through cell-resolution Zn^2+^ imaging studies in living pancreatic islets.

## Results and discussion

To develop an expedient, modular approach to targeted sensors we decided to build on the success of sensor **1**, the synthesis of which employed a high yielding Cu-mediated alkyne–azide cycloaddition (CuAAC) reaction, often simply called the “click” reaction.^21,22^ The generality of this reaction, its operational simplicity and the availability of suitable alkyne and azide starting materials has rapidly elevated it to a method of choice for the synthesis of complex non-natural molecular targets^23^ since it was first disclosed independently by Sharpless and Fokin, and Meldal in 2002. With a view to increasing the operational simplicity still further, we decided to develop a one-pot iterative methodology in which the targeting unit and the metal binding moiety are combined sequentially with a readily available fluorophore core.

### Development of a click-S_N_Ar-click approach for the synthesis of targeted probes for Zn^2+^

One-pot iterative click approaches have previously been developed that exploit the differential reactivity of dissimilar azide or alkyne functionality,^24^ or in which the second alkyne or azide component is formed *in situ.* ^25^ The latter typically rely on the use of masked alkyne units such as silyl-protected acetylenes,^26^ or masked azides in the form of leaving groups at sp^3^ centres.^27,28^ Instead we decided to focus on a novel S_N_Ar approach that makes use of the unusual reactivity of the electron-deficient naphthalimide core of **1**.^29^

Building block **2** was synthesised in high yield (96%) in one step from commercially available 4-bromo-1,8-naphthalic anhydride. We optimised our iterative approach using benzyl azide and phenyl acetylene as model substrates. Acetylene **2** readily participated in the click reaction with benzyl azide in NMP in the presence of CuI. Subsequent addition of NaN_3_ to the reaction mixture led to the S_N_Ar displacement of the bromide leaving group to give the required azide functionality for the second click reaction. The second coupling step proved more problematic. Firstly, the NaBr by-product of the S_N_Ar reaction was revealed to inhibit the click reaction in NMP, leading to slower reactions and significant levels of decomposition compared with the stepwise process. Rather than using silver salts to scavenge the bromide ions, this problem was overcome though the addition of EtOH as a co-solvent during the second click reaction, which presumably solvates the bromide anion. Secondly, even with this modification the final click reaction proved sluggish at room temperature. This was overcome by the addition of NaOAc as a base to accelerate the reaction.^30^

**Fig. 3:**
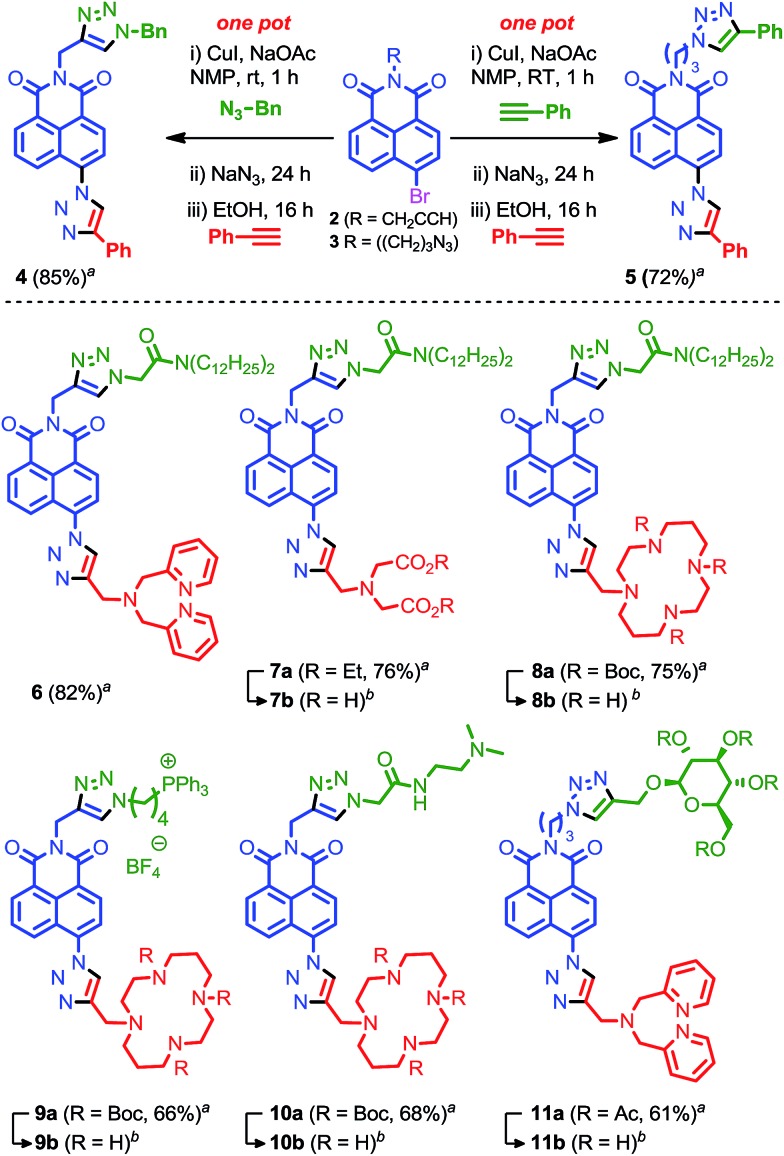
Our one-pot iterative click approach to targeted sensors for Zn^2+^. ^*a*^isolated yield from one-pot synthesis. ^*b*^For deprotection conditions and yields see ESI.†

Combining these results led to conditions under which the click-S_N_Ar-click sequence could be performed in one-pot over a 40 h period at room temperature through simple sequential addition of reagents with purification only performed after the final step (Fig. 3). Gratifyingly, in addition to being operationally trivial, and time and reagent efficient, the isolated yield of the one pot procedure significantly exceeded that of the stepwise process with model compound **4** produced in 85% yield compared with 41% with isolation and purification after each step.

With an effective iterative procedure in hand we investigated its utility through the synthesis of targeted sensors for Zn^2+^ based on metal binding units previously reported to confer a selective response to zinc^15,16^ and a selection of readily available biological targeting units. Accordingly, starting from **2** we synthesised sensors **6**–**8** that are designed to interdigitate in the cell membrane through the lipophilic di-dodecylamide motif,^13^ but in which the metal binding unit is varied between dipicolylamine (**6**), aminodiacetate (**7**) and cyclam (**8**). Having demonstrated the flexibility of our approach with respect to the metal binding unit we investigated simple variations in the targeting unit through the synthesis of sensors bearing a phosphonium salt previously demonstrated to target the mitochondria (**9**)^19*d*^ and a simple bis-amide, which has been shown to accumulate in the lysosomal space (**10**).^19*e*^

Finally, given that a wide range of propargylated biological motifs are available, in particular propargyl glycosides,^31^ we extended our iterative approach to azide functionalised building block **3** which is readily synthesised from 4-bromonapthalic anhydride (2 steps, 79% overall yield) and evaluated its behaviour under our optimised to conditions. Pleasingly, **3** was converted to model compound **5** by iterative one-pot reaction with two portions of phenyl acetylene in excellent yield. Using these conditions, sensor **11**, which bears a representative protected glycoside, was produced in excellent yield, further extending the utility of our iterative click approach.

All sensors were isolated in good to excellent yield over 3 steps; 61–82% overall, which corresponds to an average of 85–92% for each chemical step. These results clearly show that our iterative method can be used to prepare sensors efficiently and rapidly by combining biological targeting groups bearing either an alkyne or an azide with fluorescent cores **2** or **3** respectively, and metal binding domains functionalised with an acetylene handle.

### Fluorescent properties of our targeted Zn sensors

**Fig. 4:**
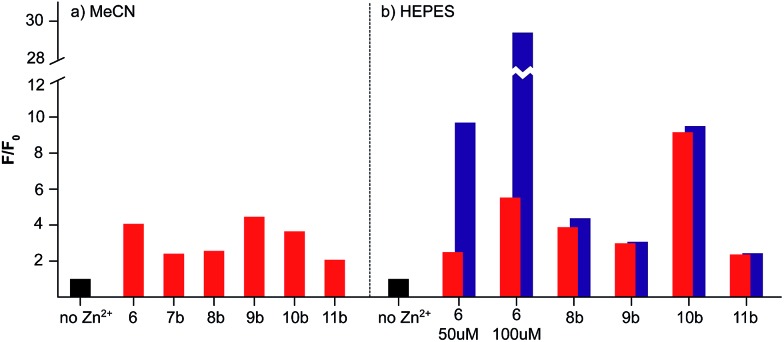
Fluorescence switch-on response to Zn^2+^ of sensors **6**–**11** in (a) MeCN and (b) HEPES buffer. Emission intensity (*F*) in the presence of **1** (red) or **5** (blue) eq. Zn^2+^ relative to the emission intensity of the sensor alone (*F*_0_, black, normalised to **1**).

The combinations of metal binding unit and fluorophore in sensors **6**–**11** are based on previously reported selective sensors for Zn^2+^ with an expected 1 : 1 binding stoichiometry. In order to confirm that our novel sensor constructs maintain these selective properties with the inclusion of a targeting unit we investigated their response to Zn^2+^ in both MeCN and HEPES buffer (Fig. 4). Where necessary, the metal binding domains and biological targeting units were deprotected using standard procedures prior to testing (see ESI† for full details).

In all cases, the sensors responded to 1 equivalent of Zn^2+^ in MeCN as expected with switch-on fluorescent enhancements of between 2- and 4.5-fold observed, and little or no further enhancement with the addition of further equivalents of Zn^2+^ (Fig. 4a).^13–15^ Similarly, with the exception of sensors **6** (*vide infra*) and **7**, which precipitated on addition of Zn^2+^, the probes responded as expected in HEPES buffer, producing similar or enhanced on/off ratios to those observed in MeCN (Fig. 4b). Dissociation constants (*K*_d_) were determined by non-linear regression analysis of titrated samples of sensors **8**–**11**, in HEPES assuming a 1 : 1 binding stoichiometry. Although reasonable values of *K*_d_ were obtained when compared to those reported for structurally similar ligands,^15r,16a,32^ the goodness of fit to a 1 : 1 binding model varied considerably (see ESI† for full details). Similar issues have recently been identified by Ceroni, Doddi and co-workers in their investigations of dendrimeric cyclam sensors for Zn^2+^,^33^ and work is currently underway to determine the origin of these effects in our systems. In summary, these *in vitro* results clearly demonstrate that the selective switch on response of probes **6**–**11** to zinc is not affected by the inclusion of a biological targeting group.

### Investigation of the anomalous behaviour of sensor **6**

**Fig. 5:**
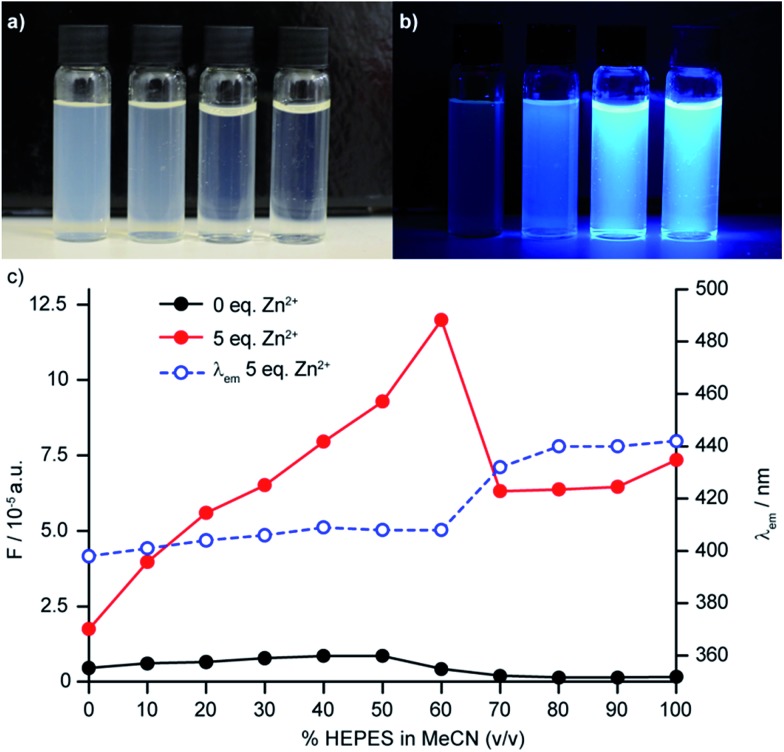
(a) Visualisation of aggregating sensor **6** at 100 μM in HEPES buffer indicating decreasing visual turbidity upon addition of excess Zn^2+^ (from L–R: 0, 1, 5, 15 eq. Zn^2+^); (b) visualisation of aggregating sensor **6** at 100 μM in HEPES buffer under UV lamp (*λ*_ex_ = 365 nm) (from L–R: 0, 1, 5, 15 eq. Zn^2+^); (c) HEPES/MeCN titration of aggregating sensor **6** demonstrating both the change in fluorescence emission and red the shift of the emission wavelength upon increasing the percentage of HEPES in MeCN.

The behaviour of sensor **6** in HEPES buffer proved more complicated than that of the other sensors investigated; addition of further equivalents of Zn^2+^ led to further significant increases in fluorescence up to a maximum value of 15-fold fluorescence enhancement with 15 equiv. Zn^2+^ (*c.f.* 5.1 for 1 equiv. of Zn^2+^) at our standard sensor concentration of 50 μM. Along with the enhanced fluorescence response, a red shift in the emission maximum was observed (23 nm and 31 nm at 1 and 15 eq. Zn^2+^ respectively). Having observed that solutions of **6** in HEPES were slightly turbid and that their visual appearance changed on addition of Zn^2+^ (Fig. 5a) we hypothesised that this unusual behaviour might be due to aggregation phenomena, driven by the surfactant-like nature (polar dipicolylamine head group, lipophilic aliphatic tail) of the sensor construct, which the addition of Zn^2+^ could serve to modulate.^34^ In keeping with this hypothesis, increasing the probe concentration to 100 μM led to an even more dramatic 35-fold increase in fluorescence intensity in the presence of 15 eq. of Zn^2+^, while at a sensor concentration of 10 μM the response to excess Zn^2+^ was reduced. Dynamic light scattering (DLS) experiments confirmed the presence of aggregates and, importantly, that the average size and polydispersity of the aggregates decreases significantly on addition of Zn^2+^ and that these aggregates remained even when probe concentration was significantly reduced to 10 μM. No such aggregation was observed in MeCN.

To examine this further, we investigated the effect of solvent composition on the fluorescence of sensor **6**, in mixtures of HEPES and MeCN.^34*b*^ In the absence of Zn^2+^, the fluorescence output and emission wavelength of **6** remained largely unchanged as the v/v% of HEPES in MeCN was varied. Conversely, in the presence of Zn^2+^ a steady increase in fluorescence output was observed up to 60% v/v HEPES, above which a significant drop in the fluorescence output was observed, which coincides with the red shift observed in the fluorescence emission (Fig. 5b).

Thus, it appears that two factors play a role in the dramatic sensitivity of sensor **6**: (i) as in all the other sensors synthesised, binding of Zn^2+^ to the ligand unit deactivates the PET quenching process leading to a “switch on”; (ii) the aggregation of the unbound sensor initially serves to quench the emission but binding of Zn^2+^ changes the aggregation such that fluorescence is recovered. Importantly, when sensor **8b** was examined by DLS, aggregation was not observed, highlighting the subtle role that small structural changes can have on sensor performance.

Finally, given the important role that aggregation plays in the performance of sensor **6** *in vitro*, and that the binding of other metal ions could possibly influence aggregation significantly leading to a loss of selective Zn^2+^ sensing behaviour, we validated the selectivity of **6** for zinc ions. Pleasingly, only Cd^2+^ and Hg^2+^ produced a fluorescent response with sensor **6**, which is to be expected based on previous studies and is not biologically relevant.^15*b*^ All other metal ions tested led either to no change in fluorescent output or quenching of the background fluorescence. Importantly, probe **6** retains its switch on response for Zn^2+^ in physiological buffer and over a physiological relevant range of pH.

### *In vitro* application of sensor 6 to monitor exocytosis *via* the release of Zn^2+^ in pancreatic islet cells

The release of insulin from pancreatic β-cells is associated with the concomitant release of Zn^2+^-containing granules, which allows the changes in the concentration of Zn^2+^ on the exterior of the plasma membrane to be used as a proxy for the release of insulin. This approach has been refined in recent work by Li and co-workers through the development of ZIMIR, a selective sensor for Zn^2+^ that anchors on the extracellular face of the plasma membrane and can be used to monitor the response of the pancreatic cells to various stimuli with high spatial resolution.^18^

The structure and *in vitro* behavior of sensor **6** bear remarkable similarities to ZIMIR in that both: (i) possess a surfactant-like structure which in the case of ZIMIR has been shown to localize the sensor to the extracellular face of the plasma membrane; (ii) display a large increase in fluorescence output in the presence of excess Zn^2+^ (70-fold in the case of ZIMIR, 35 fold in the case of sensor **6**); (iii) demonstrate selectivity for Zn^2+^ over other biologically available divalent metal ions. Given the utility of ZIMIR in monitoring the stimulated efflux of zinc, we decided to evaluate probe **6** *in cellulo* to see if it displayed similar membrane targeting behaviour.

Sensor **6** (30 μM) was incubated with pancreatic islet cells for 1 h, which were then washed under a steady flow of biological buffer for the remainder of the experiment to remove unbound sensor. In order to overcome the less favourable absorption/emission profile of sensor **6** compared with ZIMIR (*λ*_ex_ = 493 nm, *λ*_em_ = 518 nm *vs λ*_ex_ = 347 nm, *λ*_em_ = 443 nm), the cells were imaged using 2-photon excitation (*λ*_ex_ = 850 nm), which is fast-becoming the imaging modality of choice, to visualise the distribution of the **6** within the first few islet layers. After incubation and irrigation with buffer, sensor **6** remained clearly localised on the cell surface, appearing to stain the plasma membrane in a manner similar to ZIMIR with little or no internalisation of the probe over the time scale of the experiment.

**Fig. 6:**
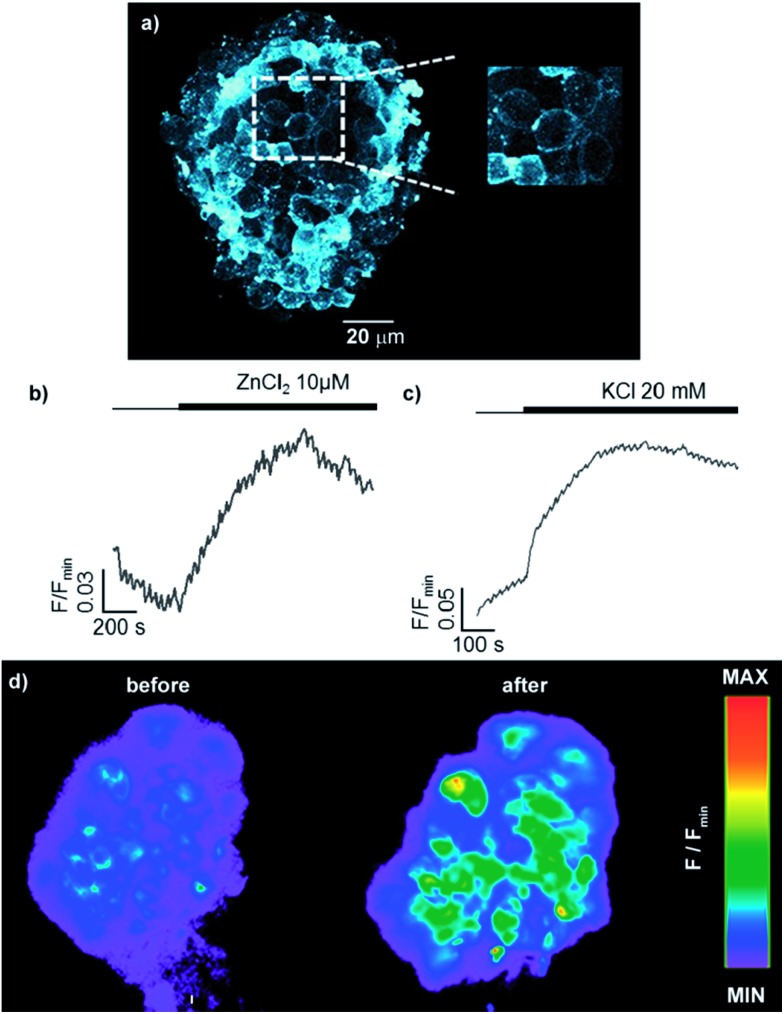
Fluorescence study of primary mouse islets of Langerhans incubated for 1 h with **6** (30 μM). (a) Two photon image (*λ*_ex_ = 850 nm, *λ*_em_ = 400–550 nm, max z-projection 80–100 μm); Response of sensor **6** over time to exogenous (b) 10 μM ZnCl_2_ and (c) 20 mM KCl measured by epifluorescence microscopy. (d) Epifluorescence images demonstrating the response to Zn^2+^ co-released with insulin from dense core secretory granules after stimulation with KCl (20 mM).

Having demonstrated that sensor **6** displays the expected cell-membrane targeting behaviour, we used epifluorescence microscopy to investigate the probe's response to changes in the extracellular concentration of Zn^2+^. Trivially, addition of extracellular Zn^2+^ led to an increase in fluorescence, confirming that **6** is indeed anchored on the extracellular face of the plasma membrane (Fig. 6b). More interestingly, when KCl was administered, to cause membrane depolarisation and release of insulin and Zn^2+^ into the extracellular space,^35^ sensor **6** exhibited a strong fluorescence response (Fig. 6c) in a manner similar to that previously observed with ZIMIR. These results suggest that readily available sensor **6** could also be used to map the release of insulin at the cellular level but has the advantage of being significantly less challenging to prepare than ZIMIR. Intriguingly, when the same experiment was attempted with sensor **8b**, despite the structurally similarity to sensor **6** (polar metal binding unit, lipophilic tail), no increased fluorescence response in the cell membrane was observed (see ESI†). This result, combined with the unexpected insolubility of the zinc complex of sensor **7**, which could also be expected to act as a membrane targeting probe, demonstrates the “trial and error” nature of sensor design for biological applications which our diversity oriented approach can facilitate.

**Fig. 7:**
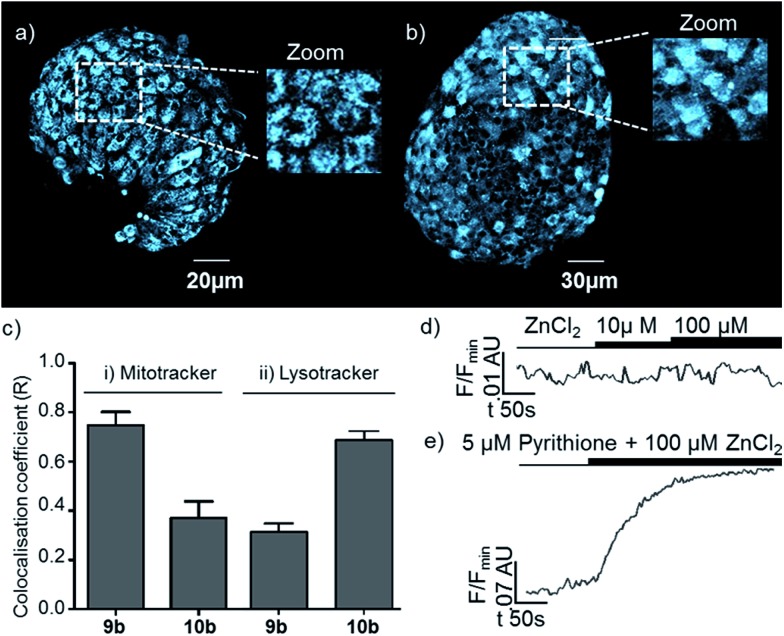
Epifluorescence study of sensors **9b** and **10b** (300 μM). Two photon image of sensor **9b** (*λ*_ex_ = 850 nm, *λ*_em_ = 400–550 nm, max z-projection 80–100 μm); (b) as for (a) but sensor **10b**. (c) comparison of co-localisation coefficients for sensors **9b** and **10b** with (i) Mitotracker and (ii) Lysotracker; (d) response over time of sensor **9b** to exogenous (d) ZnCl_2_ and (e) 100 μM ZnCl_2_ co-administered with 5 μM pyrithione.

In order to further demonstrate that the behaviour of the membrane targeting probe **6** relies on it being localised on the extracellular face of the plasma membrane, and also that probes based on our new general sensor scaffold are capable of targeting intracellular organelles, we also examined probes **9b** and **10b** which were designed to target specific organelles *in cellulo*. After incubation, the cellular distributions of **9b** and **10b** were different to that of **6**, and from one another (Fig. 6a and b): both probes displayed an extranuclear localisation with a punctate appearance consistent with their presence in or on intracellular organelles. However, the appearance of islets incubated with sensor **10b**, which is designed to target the lysosome, is qualitatively different to those incubated with mitochondria targeted probe **9b**, with **10b** localised in larger bodies. The selective and differential localisation of **9b** and **10b** was confirmed by co-localisation with Mitotracker and Lysotracker respectively (Fig. 7c).^36^ In keeping with the complete internalisation of probes **9b** and **10b**, addition of extracellular Zn^2+^ was shown to result in essentially no change in their fluorescence response (Fig. 7d). Conversely, when pyrithione, which permits transport of Zn^2+^ across the plasma membrane, and Zn^2+^ were co-administered, both **9b** and **10b** showed a significant increase in fluorescence (Fig. 7e). Finally, we were also able to show that none of the probes were cytotoxic as incubation of mouse islets with probes **6**, **9b** and **10b** does not significantly induce cell death (necrosis) compared to DMSO-alone (see ESI†).

## Conclusions

We describe here the development of a new range of targeted Zn^2+^ sensors that can be efficiently synthesised in high yield in an operationally simple manner. We anticipate that this approach will significantly accelerate the development of probes for application in biological systems. The importance of ease of synthesis in order to overcome the difficulties associated with their trial and error optimisation *in cellulo* was inadvertently exemplified through the disparate behaviour of sensors **6**–**8**, all of which were originally anticipated to be suitable for staining the plasma membrane in biological systems. Testing of sensors bearing representative targeting units in pancreatic islet cells demonstrated not only their differential localisation but also their divergent responses to extracellular stimuli, suggestive of applications in the study of Zn^2+^ trafficking in the subcellular space. In particular, sensor **6** allowed us to monitor insulin release *in vitro* in living pancreatic islet cells, using Zn^2+^ as a proxy, in a manner analogous to ZIMIR. This is suggestive of great potential in the high-resolution imaging of insulin secretion, an important goal in the study of diabetes. Thus our modular synthetic approach will facilitate the optimisation and application of targeted Zn^2+^ sensors for biology, allowing the manufacture of tailor-made probes, such **6**, with applicability for the study of Zn^2+^-signalling and insulin exocytosis.
